# Hospital and Institutional News

**Published:** 1914-01-31

**Authors:** 


### HOSPITAL AND INSTITUTIONAL NEWS.

### THE CAMBERWELL INFIRMARY LIBEL CASE.

We congratulate Dr. W. J. C. Keats, the medical superintendent of Camberwell Infirmary, on the verdict which, as we note fully elsewhere, has been awarded in his favour over the action for libel which he brought against a newspaper for statements published with reference to the corporal punishment of certain unruly convalescent boys in his institution. The jury found the statements libellous and awarded him £100 damages, which, we think, did not err on the side of generosity in view of the suggestions which were contained in the article. The judge, moreover, directed the jury to remember that the point to be decided by them was not whether corporal punishment was the proper way of dealing with refractory children in Poor-Law infirmaries. On this important question we have already expressed our views, and we return to the subject again merely for the pleasure of congratulating an infirmary superintendent on the well-deserved success which has attended his appeal to the courts in an action that must have been very unpleasant for any institutional officer of high standing to have been compelled to take. We also cannot refrain from quoting the view expressed in these columns last year at the time when the question of corporal punishment in this infirmary was under discussion. "We are afraid that the Guardians will never hear the last of it [their approval of corporal punishment] until they have found out by experience the heavy price that they are paying for condoning this practice." Have not events justified this point of view?

### THE NEW STAFF APPOINTMENTS AT CARDIFF.

On November 15 we discussed in a leading article the controversy over the suggested qualifications for future appointments at King Edward VII.'s Hospital, Cardiff, pointing out that if the Cardiff school wants the best possible teachers the proposed statute, that no one be appointed physician unless he be an F. or M.R.C.P., nor surgeon unless he be an F.R.C.S., was essential. This proposal was negatived by an adverse majority of three, and in consequence of the interest excited over the matter, the new appointments have been awaited with eagerness by everyone. They were made last week, when Mr. Ivor Jones Davies, M.D., M.R.C.P.(Lond.), M.B., B.S.(Lond.), and Mr. Herbert Thomas Evans, B.M., B.Ch.(Oxford) and M.B., B.S.(Lond.), were appointed assistant honorary physicians. Mr. Davies's distinctions include those of Glamorgan Scholar, University College, Cardiff, and Hospital Scholar, St. Bartholomew's Hospital, while he has acted as clinical assistant, with temporary charge of medical out-patients, at King Edward VII.'s Hospital, and as a clinical assistant in its pathological department. He has also held appointments at the Royal Free Hospital, St. Bartholomew's Hospital, and the Great Ormond Street Children's Hospital, London. Mr. Evans, who is an M.A. of Oxford, was educated at Christ's College, Brecon, Jesus College, Oxford, and St. George's Hospital, was formerly house surgeon at the Radcliffe Infirmary, Oxford, and has been clinical assistant at the Children's Hospital, Paddington Green. Both are understood locally to have been appointed for their qualifications for teaching, and one, at least, fulfils the letter of the rejected statute referred to above. In spite of differences of opinion, the selection committee was trusted by all parties, and the new appointments have been eagerly awaited by those who have at heart the welfare of the Welsh National Medical School.

### INSTITUTIONAL PORTERS' FINE RECORDS.

St. George's Hospital has just parted with three old and faithful porters, whose joint record, as outlined in the *St. George's Hospital Gazette*, has been in some respects unique. Mr. Trickey has been thirty-eight years in the hospital service, for twenty-five of which he was casualty-room porter. To see his bandaging was a liberal education to medical students and residents; and it is said that his capeline head bandage was absolutely irremovable otherwise than by unwinding it. He also acquired great dexterity with dental extraction forceps, and was a skilful masseur as well: truly a fine list of accomplishments. The next, Mr. Wright, has an even more extraordinary record. He served twenty-six years in the police, as well as twenty-six years at St. George's, and now retires at the age of eighty-one. His work has always involved exceedingly heavy manual labour, and to see this wonderful octogenarian mounting long flights of stairs under a burden which most men of half his age would decline to lift at all was a perpetual marvel. Last of all, Mr. Stanley retires from the post of indoor messenger, which he has filled for twenty-nine years. An institution which can keep its servants for periods such as these deserves part of the credit for their long terms of service; and it is to be hoped that the example of the three veterans will encourage their juniors to emulate them in efficiency and length of service.

### HOW A WAITING LIST MAY BE REDUCED.

Some very interesting points were made at the quarterly meeting of governors of the Leicester Royal Infirmary last week, when Mr. Harry Johnson, house governor and secretary, presented the annual report. For the first time practically 4,000 in-patients were admitted, the actual number being 3,993, meaning an average of eighty admissions a week and a rise in the number of patients received during the year of no fewer than 371. This rise is attributed frankly to the Insurance Act, which has "disclosed" many cases requiring institutional treatment. To meet the problem of an increasing waiting list it was suggested that "in ordinary surgical conditions of no urgency" patients should ask their doctors to inquire the cost of treatment at the Faire Hospital, and if they laid by their Insurance benefit and club money, in a few weeks, added the report, they would be able to pay for their treatment themselves. This course would be rendered necessary in time, and would allow severe cases to be admitted to the infirmary without waiting. As regards cases of surgical tuberculosis, provision for this having been made by the Insurance Act, it was hoped to arrange a conference with the town and county authorities urging that the provision of treatment should not be left to voluntary effort alone. Out-patients have decreased, as anticipated, and allowed this department to be "what it was really intended," a place where the sick who cannot afford a specialist can obtain advice. Annual subscriptions, donations, and church collections all show increases, and the Saturday Fund, described as the Eldorado of the hospital's income, rose by practically £400 to the splendid sum of £15,550—more than half the income required. The expenditure totalled £25,005, leaving a deficit of only £124 to be carried forward. The Children's Hospital is expected to be opened in March, and that will mean that the expenses for the year will probably reach £29,000, and it was stated that the new hospital would probably double the accommodation of the temporary building, where 470 children have been received.

### WHAT AN ANNUAL MEETING OUGHT TO BE.

In concluding a most eloquent speech, Mr. Harry Johnson said that he had kept a record of the approved societies to which in-patients belonged, and that some of them would be surprised to learn the large numbers of their members who had received treatment, and a conference with them on contributing to the hospital is hoped for. Few annual meetings can show so much life and interest as that of the Leicester Royal Infirmary, which was, as has become usual, well attended, and no one glancing at the main points in the report which we have here given, and at the manner of their presentment, will be surprised at the interest shown. The institution is what it claims to be, "the people's hospital," alive in every part of its administration and through its work on the very pulse of the district which it serves.

### PENALTIES FOR NON-NOTIFICATION.

Mr. Charles Edward Alexander McLeod, a practitioner of Notting Hill, was summoned last week for failing to nctify a case of scarlet fever, and Mr. Alfred Samson for causing a person suffering from this disease to be placed in a taxi-cab. The Kensington Borough Council issued the summonses, and their counsel stated that Mr. Samson's son, attended by Dr. McLeod, went to a convalescent home after recovering from scarlet fever. After the room had been disinfected a nurse, who cleaned it, contracted the disease. After Dr. McLeod had seen her Mr. Samson sent her to her home in a taxi-cab, where the panel doctor ordered her to the fever hospital. No notification, continued counsel, was sent by Dr. McLeod, the first intimation coming from the girl's doctor. Dr. McLeod said he did not notify the disease because he was not the girl's doctor; he was merely asked to examine her, and he concluded that her doctor would notify the case. He knew nothing of the taxi-cab incident. The magistrate fined Mr. Samson five guineas and three guineas costs, and said that it would be difficult to imagine conduct more wicked of its kind than his. He accepted the doctor's plea of not intending to break the law and fined him twenty shillings and costs. All must agree that the notification of infectious disease is so important that it is as necessary to punish errors of judgment as breaches of the law.

### THE PUBLIC AND CHILLED MEAT.

At the Clerkenwell Police Court last week a large firm of meat-sellers and an individual shopkeeper were fined for unlawfully applying the false trade descriptions of "Best English" and "Prime Scotch" to pieces of beef which were not in fact English or Scotch, contrary to the Merchandise Marks Act. The Board of Agriculture issued the summonses, and the meat was chilled meat. It is obviously undesirable that false trade descriptions should be permitted, but on the whole we doubt whether the mass of the public is much deceived by them. A good housekeeper can tell from the price charged whether a particular joint purports to be English or not, and in the cases in question it is interesting to note that no attempt was made to ask higher prices on the ground that the descriptions were correct. Opinions vary as to whether for cooking purposes chilled meat is or is not inferior, and now that "home killed" is often used to describe imported meat killed in this country it seems that, provided that the meat is good to eat, the public considers price alone, indifferent to origin.

### PARENTS AND OPERATIONS.

At a meeting of the London Education Committee last week, Mr. F. R. Anderton presented a report of the Children's Care (Central) Sub-Committee which may prove of considerable importance. It called attention to the recent judgment in the King's Bench, which had enabled the Leicestershire Education Authority to secure the conviction of a parent who had declined to allow his child to undergo an operation for adenoids. The report went on to say that though each case must be considered on its merits, similar convictions in the light of this judgment could probably be secured, especially in "simple and safe operations." It seems that the sub-committee has not yet sent any case involving the question of an operation to the National Society for the Prevention of Cruelty to Children for prosecution. In the discussion which followed, Mr. Bruce expressed the view that the matter was so delicate that the Council itself should be directly responsible for any action that was set on foot. Mr. Anderton said that so long as he was chairman he should be very slow to suggest any prosecution over the question of operations. The views of the sub-committee were then endorsed. The matter is one that can only be decided practically in each particular case which may arise. Who would care to define a simple and easy operation, since not merely the surgery but the anæsthesia may meet with difficulties and dangers unforeseen? The greatest caution is necessary, and we are very glad to see that the committee appeared to realise this without any dissentient voice.

### THE SATURDAY FUND AWARDS.

At a special meeting of the Board of Delegates of the Hospital Saturday Fund last Saturday, over which Sir Savile Crossley presided, the recommendation of the Distribution Committee to award £28,400 odd among 214 institutions was decided on. Three fewer institutions thus participate than was the case in 1912, and the grants were £4,081 lower than on that occasion. It will be noted, therefore, that the awards have had to be curtailed in amount, but that, practically speaking, the same number of institutions benefit. We have previously noted the fact that the total receipts, £40,309, are £5,009 less than in 1912. Sir T. Vezey Strong is to preside at the annual dinner, which will take place on February 14, and the first meeting of the reconstituted Board of Delegates for 1914 will be held a week later—namely, February 21.

### THE SOUTH SHIELDS GUARDIANS AND THE USE OF ALCOHOL.

In a letter to the *Shields Daily Gazette and Shipping Telegraph* the Rev. J. Gill, the vice-chairman of the South Shields Board of Guardians, refers to our recent criticism of that Board's action in refusing the requisition of the medical officer for whiskey, and denies that the Board acted illegally in doing so. He states that what the Board did was "to put aside the request for some considerable supply of whiskey in advance of immediate necessity." He adds that, so far as he was concerned, "it was not denied that a doctor must use such treatment as seems to him most beneficial for his patients." The newspaper reports, however, show that some of the members did deny this right to the infirmary doctor, and that the resolution moved and carried was that further requisitions for intoxicating liquors should be refused. This is undoubtedly an illegal resolution, and no good intentions on the part of individual members can alter its plain meaning and its implied menace to the medical officer or make it legal. Mr. Gill, in his letter, makes some mis-statements, and falls into a common error in logic by deducing a general conclusion from insufficient facts and false analogies. A gallon of whiskey is not an "unnecessarily large stock" for an infirmary with over 350 beds and about 2,000 annual admissions, and the use of alcohol in most large hospitals has not "almost reached a vanishing point." That alcohol is not prescribed so freely by medical men as was once the case, and that some medical men disapprove of its use, are undoubted facts; but most medical men still regard alcohol as a most valuable and indispensable drug in many conditions of disease. This is especially the case with patients who have been accustomed to use it freely or in excess whilst in health. Such patients form a notable proportion of the inmates of most Poor-Law infirmaries. To support his case against alcohol as a drug, Mr. Gill makes use of the diminished employment of cocaine on account of its risks as an analogy, and of the evils caused by an excessive use of alcohol. Both arguments are fallacious. Cocaine is not used so much now as formerly because other drugs of a similar character, but comparatively free from danger, are now available. And if alcohol is not to be used in proper doses because its excessive use is to be condemned, then on similar reasoning we must abandon the use of opium, strychnine, and most other drugs, tea, and even food.

### DAMAGES AGAINST A NURSING HOME PROPRIETOR.

A good deal of sympathy is being expressed with Dr. Bradford, who has been cast in damages of £100 owing to accidental burns sustained by a patient in the nursing home which the former conducts. It was obvious on the evidence that liability for negligence would attach to someone over the matter; and the chief point of interest of the case to managers of private hospitals lay in the decision as to whose the liability might be. The nurse whose act resulted in the burns was not brought into the action by either side. The plaintiff sued the proprietor on the ground that a contract of service existed between them, and that the nurse was an employee of Dr. Bradford's, for whose acts he was responsible. The defence was that in doing what she did the nurse was carrying out the direct orders of the surgeon, which were not conveyed through Dr. Bradford at all. The correctness of this statement of fact appears to have been admitted, but it did not avail to shift the burden of responsibility on to the shoulders of the surgeon. Technically, there is little doubt that the verdict was just and proper; but, all the same, it is hard lines on Dr. Bradford, whose project of a big private hospital of two hundred beds was commented on in The Hospital only a fortnight ago. The solicitors who prepared his case are those whom the London and Counties Medical Protection Society retains: so probably this society has sustained one of its very infrequent rebuffs.

### CO-OPERATION IN PUBLIC HEALTH WORK.

In a recent letter to the Mayor of each of the Metropolitan Boroughs and the Chairman of each of the Metropolitan Boards of Guardians, the Local Government Board suggest that a conference should be held with representatives of the voluntary associations engaged in public health work with a view to formulating some scheme of co-operation. This is a step in the right direction and may lead to very valuable results. It is to be hoped that the conferences will give rise to an extension of the help already given one another by many of the Metropolitan Borough Councils and Boards of Guardians. If this is to be the case the medical officers of health and the medical superintendents of the local Poor-Law infirmaries should be included amongst the members of the conferences. There are two directions in which such mutual help might be increased. The medical officers of health might be given greater facilities for sending to the Poor-Law infirmaries patients whom it is desirable from a public health point of view should be under medical care in public institutions, and more of the pathological and bacteriological work required in Poor-Law infirmaries might be undertaken by the Public Health Departments. Some of the Metropolitan Borough Councils already undertake the bacteriological examination of swabs and sputa sent to them by the medical practitioners in their areas. When this is the case the local Poor-Law infirmaries usually take full advantage of the facilities thus afforded. A few Borough Councils go further and undertake the examination of cerebro-spinal fluid and pathological exudates, and at least one has recently arranged for the examination of blood for the Wassermann reaction. It is highly desirable that similar arrangements should be made in all the Metropolitan boroughs.

### A CENTRAL LABORATORY FOR LONDON INFIRMARIES.

The system should be extended so that the councils would provide for the pathological, chemical, and bacteriological examination of all morbid products obtained from patients sent to them by the medical officers of voluntary and Poor-Law institutions. The advantages to the cause of public health and to those institutions the staff of which does not include a skilled pathologist and bacteriologist are obvious. In the case of Poor-Law institutions the Local Government Board, however, have a direct personal responsibility, and it is high time they took the matter into their own hands. They possess a highly skilled staff and excellent laboratories at present engaged chiefly in research work. It would be easy to arrange by an increase of this staff for all the pathological work of the Poor-Law infirmaries in and near London to be performed in a central laboratory under the Board's control. A scheme of this kind would have the further advantage that facilities could be afforded to the medical staff of the various infirmaries to carry out themselves routine and research pathological work in this laboratory.

### NEW TREASURER AT SALFORD ROYAL HOSPITAL.

Dr. Edward Hopkinson has been appointed treasurer to the Salford Royal Hospital in succession to the late Mr. Frederick Burton, who was elected a member of the board in 1886 and became treasurer in 1898, an office which he held, to the great advantage of the institution's investments, till his death. Dr. Hopkinson, who has been a member of the hospital's board for many years, has also acted as chairman of the building committee.

### A WINTER IN AN ICE-CAVE.

On Monday last, at the Royal Society of Medicine, Surgeon Murray Levick gave an account of some of the experiences of Captain Scott's northern party from a medical point of view. The lecture was listened to by a very large audience, and was freely illustrated by superb photographs of those terrible yet magnificent icy wastes. It was obvious that the lecturer's account of the health of the party of six, who spent seven months in a very confined space hewn out of the solid ice underfoot, appealed very strongly to the audience of fellows and members. According to the teaching of physiology none of the party should have been able to exist in this igloo, which measured but six feet by twelve and had a sloping roof from three to five and a half feet high. At night all was hermetically sealed, even the tiny chimney; and although the amount of oxygen was insufficient to allow of the combustion of a blubber lamp or even of a match, the lecturer and his companions experienced no respiratory discomfort while lying in their sleeping-bags. It is certain that the dietary experiences of the party will, when more fully reported, receive careful study at the hands of the physiologists, who have here fruitful and exceptional material to investigate. The trials of the little band from diarrhœa, uric acid, and ptomaine poisoning were probably harder to bear than any of their physical fatigues and the consequences of cold. The long-drawn-out pain of anal fissures and hæmorrhage was enough to take all the spirit out of the pluckiest. An interesting but, under the circumstances, a most serious affection was that of enuresis, which afflicted the members of the party after some weeks of a diet of steel-hard meat. Seeing that normally it was a work of an hour or more to put on the stiffly frozen clothes, such an added trial made their sufferings a subject better imagined than described. A cure or a relief was obtained by boiling thoroughly the meat. In spite of their diet and environment it is a remarkable fact that no one suffered from scurvy; this the lecturer ascribed to the carnivorous diet.

### DEATHS UNDER ANÆSTHETICS.

During an inquest at Southwark last week on a man who died under an anæsthetic at Guy's Hospital, Dr. F. J. Waldo, the Coroner, said that he had seldom now to investigate deaths under anæsthetics from Guy's, whereas formerly he had inquired into as many as eleven in one year. How did the doctors account for it? Dr. Thomas Bennett, house physician and anæsthetist at Guy's Hospital, said that he believed it was due to the remarks made by Coroners' juries that only specially trained anæsthetists should be employed. The witness said that much harm was done to anæsthetists from the reporting of such cases in the lay press, whereupon the Coroner said that much valuable matter was wasted in Coroners' Courts where so much expert medical evidence was given nowadays. The medical press might report more cases of this sort than they did, and he was pleased to give it and other pressmen every opportunity. The witness went on to say that proper records were now kept at Guy's, and admitted that good had been done by the Coroners' inquiries, and even by the publicity which ensued. Dr. Bennett further explained that the practice now at Guy's was for twelve visiting specialists to give anæsthetics, and in their absence four house physicians took their places. In the case in question he gave chloroform and ether mixed and oxygen to the deceased. All three doctors, including Dr. Spilsbury, who made an independent *post-mortem* examination, agreed that death was due to heart failure accelerated by the anæsthetic, and a verdict was returned in accordance with the medical evidence, stating that the anæsthetic was given with skill and care. The Coroner stated that a full public inquiry into all such deaths was in the true interests of both the relatives and the hospitals, and added that he always recorded on the inquisition full details concerning every such death. We agree with Dr. Waldo, and our readers will note from this case how much good publicity and the recommendations of Coroners' juries have already accomplished.

### MEDICAL BOOKS AT POOR LAW INFIRMARIES.

The Edmonton Board of Guardians recently discussed a recommendation of the Infirmary Committee that £10 annually should be spent on medical periodicals and books of reference for the use of the infirmary medical staff. The recommendation was adopted only after a prolonged discussion, and the word "annually" was deleted. The original resolution was, however, a very modest and reasonable one. Provision for the formation of a reference library of books on nursing has been made by many Boards of Guardians for the use of their probationer nurses, and a few Boards have extended the principle and provided a reference library of medical books for the medical staff. The main argument used against the suggestion at Edmonton was that the books should be bought by the medical officers themselves. It should be remembered, however, that, with the exception of the medical superintendent, the medical officers are constantly changing, and that the institutional library is meant to supplement, and not to replace, the private libraries of the staff. Most medical men in London make use of the libraries of the Royal College of Physicians, the British Medical Association, the Royal Society of Medicine, and others for consulting books of reference, but it is an obvious and time-saving advantage to an institution to have the most important books of reference on the premises. A medical library is as necessary a part of the equipment of a well-organised infirmary as are surgical instruments and medical appliances, and it is as much the duty of the governing body to supply the former as the latter.

### RESIGNATION OF AN OLD TREASURER.

Mr. E. H. Bousfield, who has acted as treasurer to the London Orphan Asylum for fourteen years, and has been a member of the board of management for so long a period as forty-four years, has sent in his resignation. In the meantime Mr. A. P. Blathwayt has consented to undertake the duties of treasurer until the next general court. It is unfortunate that the centenary appeal of the past year did not meet with the success which was hoped for, but the annual dinner, presided over by Prince Arthur of Connaught, realised the fine total of £20,000.

### THE CHELSEA AND SOUTHWARK DISPENSARY SCHEMES.

Of the two complete schemes for the dispensary treatment of tuberculosis which have so far been submitted to the London County Council those from the boroughs of Chelsea and Southwark are of special interest, as they are based on two different methods of providing the desired treatment. In the case of Chelsea it is proposed to establish a dispensary in connection with a hospital; in the case of Southwark it is intended to set up a municipal dispensary, and thus there will be an early opportunity of comparing the merits of the two methods. The Chelsea proposal is to establish a dispensary at the Brompton Hospital for the joint use of the inhabitants of Chelsea and of the southern part of Kensington, the hospital agreeing to appoint as tuberculosis officer a junior member of the visiting staff, who will co-operate with the medical officer of health of Chelsea. The patients are to include insured persons recommended by the medical officer of the Insurance Committee or by a local panel practitioner, and uninsured persons recommended by medical practitioners or by the borough medical officer of health. The hospital, as a tentative arrangement for six months, is to treat all patients coming under these categories at a charge of 2s. per patient per attendance. Home visiting will be carried out by the borough medical officer. The arrangements have proved acceptable to the Local Government Board, which suggests that at the end of six months a fixed annual sum should be substituted for a fee for each attendance. The charge of 2s. for each attendance at the dispensary is stated by the authorities of the Brompton Hospital to be based upon the actual experience gained during the past year. If the estimate of the Borough Council is realised the number of attendances at the dispensary will be four thousand a year, and the annual expenditure £550, of which £133 will come from the Insurance Committee, £208 from the Local Government Board, and £208 from the rates.

### A MUNICIPAL DISPENSARY AND GUY'S HOSPITAL.

The proposal of the Southwark Borough Council is to institute a municipal dispensary which it is intended to link up with Guy's Hospital. The dispensary would be under the control of a sub-committee of the Borough Council, on which six other persons would be co-opted, the medical officer of health of the borough being the chief executive and examining officer. A specially trained tuberculosis officer would be in charge of the dispensary at a salary of £500 a year. While under the control and supervision of the medical officer of health, as far as administrative duties are concerned, he would be fully responsible for the clinical side of the work. The dispensary would provide treatment both for insured and uninsured persons, act as a clearing house, and as a means for the better special education of medical practitioners in the borough. With this object practitioners would be given opportunities to attend the dispensary without fee. The estimated annual cost of this scheme is £1,152, to be provided by the Insurance Committee, the Local Government Board, and the rates in equal amounts. Arrangements are to be made for utilising Guy's Hospital for the purposes of consultation and treatment in special cases. Both these schemes meet with the approval of the Public Health Committee of the London County Council, and their early adoption would be of the greatest assistance to that Committee in framing its complete and comprehensive scheme for dealing with the treatment of tuberculosis in the county of London.

### LEPROSY IN AUSTRALIA.

The interest that Australia, from its proximity to Asia, is compelled to take in leprosy, writes our Australian correspondent, has lately received extra stimulus from several happenings. Early last year three lepers—a white man, a Japanese, and a half-caste aboriginal—escaped from Peel Island Lazaret, Brisbane. The half-caste was speedily recaptured; after two months in hiding the European surrendered and was returned to Peel Island; but the Jap got away to the East. Early in June Dr. C. R. Maitland Pattison, who had spent some years on Robben Island, Cape Town, passed along our coasts, sent by the Colonial Office to take charge of Mekagoi, a station about fifty miles from Suva, Fiji. Asked by the usual interviewer what precautions he took, Dr. Pattison said: "Hardly any. Only the observance of usual health rules." Later, in June, Mr. Wellesley Bailey, superintendent of the Mission to Lepers in India, which conducts twenty-seven homes for the untainted children of leprous parents, lectured here, with tremendous statistics of our leprous neighbours' numbers. But what has roused most horror is the fact, well known, though not published, that within the last few months two European ladies have been sent to the lazaret at Sydney. Both are wealthy and well known, the latest sufferer a young wife with an infant. Both had been travelling in the East, and the infection of the elder is set down as due to a sable coat. The latest development is hopeful—the release of three coloured men from Peel Island as cured. They have been at the lazaret for from seven to three years, and are now declared to be wholly free from the disease. It is now an accepted fact that rats in Australia are subject to leprosy (as well as bubonic plague), a conclusion arrived at by Dr. R. J. Bull, Melbourne University, a few years ago from the examination of a rat supposed to be suffering from mange.

### THE INSURANCE ACT AND PHARMACEUTICAL ORGANISATION.

As a result of the changes brought about by the operation of medical benefit under the Insurance Act, it has been necessary to remodel the Pharmaceutical Society's system of local organisation. The Society's Council recently held a conference with delegates from a hundred and ten counties and county boroughs in England and Wales for the purpose of determining what scheme of organisation is best suited to the new conditions. After lengthy consideration had been given to the matter, it was decided that a committee should be formed consisting of six members of the Council of the Pharmaceutical Society to be appointed by the Council, seven panel chemists to be elected by delegates from local Associations, and two persons to be appointed by limited companies trading as chemists. This committee of seventeen is to be known as the Pharmaceutical General Committee on Insurance, and to it will be referred matters arising out of the Pharmaceutical Insurance Service. Periodical conferences of delegates will be held for the purpose of deciding the main principles to be followed by the General Committee. By means of this new machinery it is hoped to remove some of the difficulties which stand in the way of the smooth administration of the Pharmaceutical Service. It is interesting to note that this is the first time there has been such an alliance between local Associations and the Pharmaceutical Society.

### THIS WEEK'S DRUG MARKET.

Business has been much more active and there seems to be some reason to hope that the undoubted improvement that has set in will be maintained for some little time. Since last report a considerable business has been done in menthol at much higher rates, but at the moment the market is quiet, although steady; Japanese oil of peppermint also advanced in sympathy. Offers of new season's cod-liver oil are now being made, the price being above that recently quoted for last season's oil; the Norwegian cod-fishing season is not sufficiently far advanced for an estimate to be made of the probable results, but in a week or two figures should be available from which it should be possible to form an opinion, and buyers should therefore watch the market carefully. A fair amount of business has been done in quinine, and a further advance in makers' prices at an early date would not come as a surprise. Opium is practically unchanged in price. Oil of star aniseed is slightly dearer, as also is oil of cassia. The price of bromides is unchanged and the demand is quieter. At the recent public sale of drugs in Mincing Lane there were no buyers for the larger proportion of the drugs offered and the auction passed off quietly, with few, if any, price alterations of importance to note.

### FREE SEASON TICKETS FOR THE SCOTTISH EXHIBITION.

In regard to the Scottish Nursing Conference and Exhibition to be held at Glasgow from February 7 to 11 we shall be willing to supply our readers, free of charge, with a season ticket of admission. All readers, therefore, who think of attending and wish to have the convenience of a season ticket should apply without delay to the Manager, The Hospital, 28 and 29 Southampton Street, Strand, W.C.

